# Anatomical Classification and Staging Systems of Borderline Resectable and Locally Advanced Pancreatic Cancer—A Subgroup Analysis of the NORPACT-2 Trial

**DOI:** 10.1245/s10434-025-17527-y

**Published:** 2025-06-03

**Authors:** Jacob Ghotbi, Ingvild Farnes, Dyre Kleive, Caroline Verbeke, Aart Issa Epe, Bjarte Fosby, Pål-Dag Line, Knut Jørgen Labori

**Affiliations:** 1 Department of Hepato-Pancreato-Biliary Surgery, Oslo University Hospital, Rikshospitalet, Oslo, Norway; 2 Institute of Clinical Medicine, University of Oslo, Oslo, Norway; 3 Department of Pathology, Oslo University Hospital, Rikshospitalet, Oslo, Norway; 4 Department of Radiology, Oslo University Hospital, Oslo, Norway; 5 Department of Transplantation Medicine, Oslo University Hospital, Oslo, Norway

## Abstract

**Background:**

This study aims to provide a detailed understanding of resectability and prognosis within anatomical subgroups of borderline resectable pancreatic cancer (BRPC) and locally advanced pancreatic cancer (LAPC) on the basis of established classification systems.

**Patients and Methods:**

Patients with BRPC/LAPC, defined by National Comprehensive Cancer Network (NCCN) criteria, were prospectively included from 2018 to 2020. BRPC was subcategorized by vascular involvement and LAPC by the Louisville (Lv) classification system, and both cohorts were reclassified according to the Dutch (DPCG) criteria. NCCN-defined primary resectable pancreatic cancer (PC) cases that met DPCG-BRPC criteria were included in the analysis.

**Results:**

In total, 228 patients (96 NCCN-BRPC, 92 NCCN-LAPC, and 40 reclassified from NNCN primary resectable PC to DPCG-BRPC) were included. NCCN-BRPC exhibiting both venous and arterial involvement had a lower resection rate (odds ratio (OR) 0.22, *p* = 0.038). Isolated vein involvement and baseline cancer antigen (CA)19–9 levels < 500 kU/L predicted resectability (OR 5.99, *p* = 0.005) and survival (hazard ratio (HR) 0.47, *p* = 0.024). DPCG-BRPC demonstrated higher resectability rates (67.4% versus 46.9%, *p* = 0.004) and fewer vascular resections (37% versus 58%, *p* = 0.031) compared with NCCN-BRPC. While the NCCN only predicted resectability, DPCG also predicted survival. No patients with Lv type IIIc2–4 (nonreconstructable invasion of the portomesenteric vein combined with arterial involvement) underwent resection, and this subgroup had worse survival (HR 2.08, *p* = 0.021).

**Conclusions:**

Variations within established classification systems for BRPC/LAPC impact prediction of survival and resectability. A more detailed understanding of the anatomical subgroups in BRPC and LAPC, alongside CA19–9 levels, could enhance patient stratification regarding tumor resectability and neoadjuvant strategies.

**Supplementary Information:**

The online version contains supplementary material available at 10.1245/s10434-025-17527-y.

The accurate classification and staging of patients with pancreatic cancer directly influence treatment strategies and outcomes.^1^ For patients with borderline resectable pancreatic cancer (BRPC) or locally advanced pancreatic cancer (LAPC), surgical resection remains a crucial factor for improved survival. Several studies have demonstrated that the anatomical staging criteria for BRPC and LAPC established by the National Comprehensive Cancer Network (NCCN) are predictive of surgical resectability but do not define distinct prognostic subgroups.^2–4^ The population-based Norwegian Pancreatic Cancer Trial-2 (NORPACT-2) found that patients with BRPC undergoing primary chemotherapy were more likely to undergo resection than patients with LAPC, with resection rates of 47% and 13% respectively (*p <* 0.001).^4^ However, the fourfold higher resection rate did not lead to a significant impact on overall survival. Cancer antigen (CA)19–9 > 500 or gemcitabine monotherapy as primary chemotherapy regimen were found to be associated with poor survival. These findings are in line with a recent transatlantic study that found that staging of patients with localized pancreatic ductal adenocarcinoma (PDAC) at diagnosis should be based on anatomy, CA19-9, and performance status.^5^

The NCCN classification defines three categories of resectability (resectable, borderline resectable, and locally advanced) on the basis of the extent of vascular invasion. This classification is used to recommend various treatment approaches and to stratify patients in clinical trials. Other anatomic classification systems that have been proposed include those by the Dutch pancreatic cancer group (DPCG), the Louisville group, and the Johns Hopkins group.^6–9^ While the NCCN classification has been validated in several studies, external validations of the other classification systems are scarce. The main objective of this study was to assess the accuracy of the NCCN classification of BRPC and LAPC, as reported in NORPACT-2, compared with the DPCG and Louisville group staging systems in predicting the likelihood of surgical resection and their impact on survival rates. In addition, the study aimed to predict the probability of undergoing surgical resection on the basis of various factors, including anatomy, biology, and chemotherapy regimen in patients with BRPC and LAPC.

## Patients and Methods

### Study Population, Study Design, and Treatment

Consecutive patients with biopsy-confirmed BRPC and LAPC referred to the multidisciplinary team (MDT) meeting at Oslo University Hospital between 1 January 2018 and 31 December 2020 were prospectively included. The study protocol was approved by the Regional Ethical Committee (REC Nord 2017/1382, NORPACT-2) in August 2017. Patients were classified as having BRPC or LAPC on the basis of the National Comprehensive Cancer Network (NCCN) criteria (version 2, 2017) with an experienced abdominal radiologist and two of the authors (I.F. and K.J.L.).^10^ Patients were offered participation in NORPACT-2 following written informed consent. The diagnostic work-up, treatment sequence, and surgical and medical interventions were performed in accordance with the national guidelines, as previously described.^4,11^ Chemotherapy was administered to eligible patients, with FOLFIRINOX as the preferred regimen. However, the final chemotherapy regimen was decided by the treating oncologist at the patient’s local hospital. Changes in tumor during chemotherapy were described using the modified Response Evaluation Criteria in Solid Tumors (RECIST; version 1.1).^12^ For BRPC, exploration was recommended after 2 months of chemotherapy in cases of stable disease or response and for LAPC after 4 months in case of response. Restaging was performed using a computed tomography scan of the chest and abdomen. To obtain a comprehensive dataset on patients with BRPC, according to the Dutch classification system, we conducted a retrospective data collection of patients with NCCN primary resectable pancreatic cancer within the same study period. Computed tomography scans of these patients were classified by experienced abdominal radiologists and one of the authors (J.G.), and patients who met the Dutch BRPC criteria and were explored or resected were identified. Written informed consent to use clinical information for research purposes was obtained from all these patients.

### Outcome Measures

All variables included in the baseline and treatment characteristics were recorded at time of diagnosis. The primary endpoints of interest were resection rates and survival for three classification systems: the NCCN, the DPCG, and the Louisville system.

### Risk Matrix Model

On the basis of the findings in the recently published NORPACT-2 trial,^4^ the following risk factors were included in the risk models: tumor classification according to the NCCN (BRPC, LAPC), baseline CA19-9 (< 500 kU/l, ≥ 500 kU/l), and primary chemotherapy regimen (FOLFIRINOX, gemcitabine/nab-paclitaxel, gemcitabine).

### Definitions

BRPC and LAPC were classified according to the NCCN criteria^6^ at the time of diagnosis. Retrospectively, they were classified according to the Dutch system,^7,13^ and LAPC was also classified using the Louisville classification system (Table 1).^8^

**Table 1:** Definitions of pancreatic cancer categories, according to the NCCN, DPCG, and Louisville classification systems^7,8,13^

	Celiac axis	Common hep artery	SMA	SMV/PV	Cava	Aorta
Resectable	**NCCN**: no contact **DPCG**: no contact	**NCCN**: no contact **DPCG**: no contact	**NCCN**: no contact **DPCG**: no contact	**NCCN**: ≤180° tumor contact & no contour irregularity **DPCG**: ≤90° contact	**NCCN**: no contact	**NCCN**: no contact
Borderline Resectable	**NCCN**: ≤ 180° contact **DPCG**: ≤ 90° contact	**NCCN**: reconstructable involvement **DPCG**: ≤ 90° contact	**NCCN**: ≤ 180° contact **DPCG**: ≤ 90° contact	**NCCN**: ≤180° tumor contact with contour irregularity, or >180° contact **DPCG**: >90-270° contact	**NCCN**: solid tumor contact	**NCCN**: no contact
Locally Advanced	**NCCN**:>180° contact **DPCG**: >90° contact	**NCCN**: unreconstructable involvement **DPCG**: >90° contact	**NCCN**: >180° contact **DPCG**: >90° contact	**NCCN**: unreconstructable involvement **DPCG**: >270° or occlusion		**NCCN**: solid tumor contact
Louisville system for LAPC	**Lv type IIIa:** Encasement of the CT—with/without reconstructable PV/SMV-involvement		**Lv type IIIb1:** Encasement of the SMA—with/without reconstructable PV/SMV-involvement. **Lv type IIIb2:** Encasement of the SMA *and* CT—with/without reconstructable PV/SMV-involvement	**Lv type IIIc1:** Non-reconstructable invasion of the PV/SMV *without* arterial involvement **Lv types IIIc2-4:** Non-reconstructable invasion of the PV/SMV *with* arterial involvement		

Significant values are highlighted in bold

### Statistical Analyses

Data analyses were performed using STATA version 18. Categorical variables are expressed as frequencies and percentages and compared using Pearson’s chi-squared test. The student’s *t*-test and Mann–Whitney *U* test were used for continuous variables, depending on normality of the distribution. Comparisons of more than two continuous variables were conducted using one-way analysis of variance (ANOVA) and the Kruskal–Wallis test, depending on normality.

Univariate and multivariate logistic regression models were employed to identify independent factors for resection. Variables with a *p* value < 0.05 in univariate analyses were included in the multivariate models, excluding those with high correlation and groups with less than ten observations. In the LAPC cohort, three variables (Louisville type, chemotherapy type, and RECIST) showed complete or quasi-complete separation.^14^ For these variables, different models were tested, including the Exact method, Firth’s logistic regression, and exclusion from the analyses. The Exact method of logistic regression provided the best fit.

**Fig. 1:**
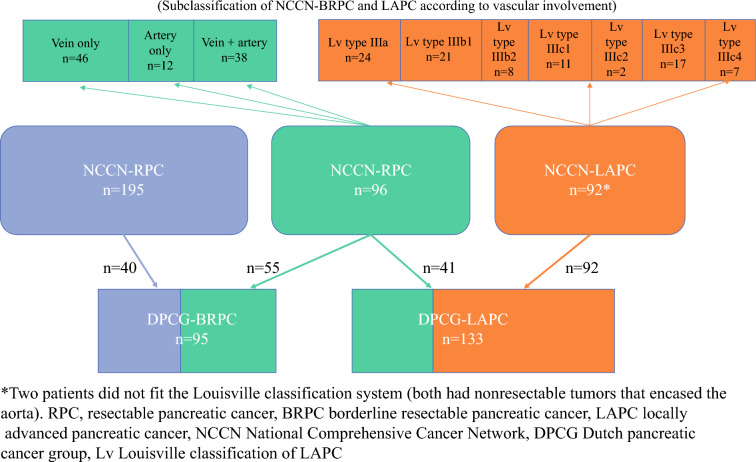
Distribution of patients based on the NCCN, DPCG, and Louisville classification systems

Overall survival (OS) was measured from the time of diagnosis until death or the last follow-up. Final follow-up was carried out on 1 January 2023. Crude differences between groups were assessed using the log-rank test and the Kaplan–Meier method. Univariate and multivariate Cox proportional hazards regression models were used to identify potential independent prognostic factors for survival. Variables with a *p* value < 0.05 in univariate analyses were included in the multivariate models. Statistical significance was set at *p* < 0.05. The 95% confidence intervals (CIs) already listed in the tables have not been included in the text Fig. 1.

## Results

**Fig. 2:**
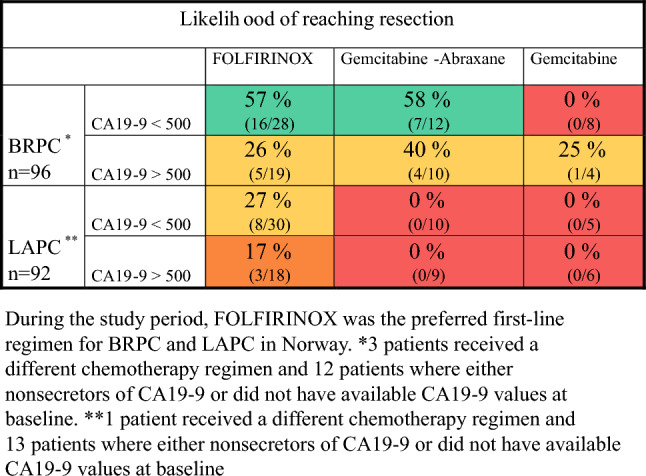
Risk matrix model depicting the probability of surgical resection in NCCN-BRPC and NCCN-LAPC

Between 1 January 2018 and 31 December 2020, our institution evaluated a total of 383 patients with localized pancreatic cancer who were eligible for either upfront surgery or primary chemotherapy. Of these patients, 195 (50.9%) were diagnosed with primary resectable pancreatic cancer (PC), 96 (25.1%) with BRPC, and 92 (24.0%) with LAPC. The 96 patients with BRPC and 92 patients with LAPC were prospectively enrolled in the NORPACT-2 study, while the 195 patients with primary resectable PC were retrospectively reviewed to identify those who could be reclassified as BRPC on the basis of the DPCG criteria. The baseline characteristics of the 40 patients who were reclassified are detailed in Supplementary Table 1, while those of the patients with BRPC and LAPC are presented in Supplementary Table 2 and 3, respectively. As recently published by our group, the resection rates were 45 of 96 (46.9%) for BRPC and 12 of 92 (13%) for LAPC, and the median OS rates for patients that underwent resection were 27 months and 33.2 months, respectively (hazard ratio (HR) 1.22, *p* = 0.224).^4^ For the total cohort of BRPC and LAPC (*n* = 188), baseline CA19-9 > 500 kU/L both predicted lower resectability (odds ratio (OR) 0.42, *p* = 0.001) and impaired survival (HR 1.82, *p* = 0.001). The risk matrix model in Fig. 2 illustrates the likelihood of reaching resection in our cohort.

### Borderline Resectable Pancreatic Cancer (BRPC)

**Fig. 3:**
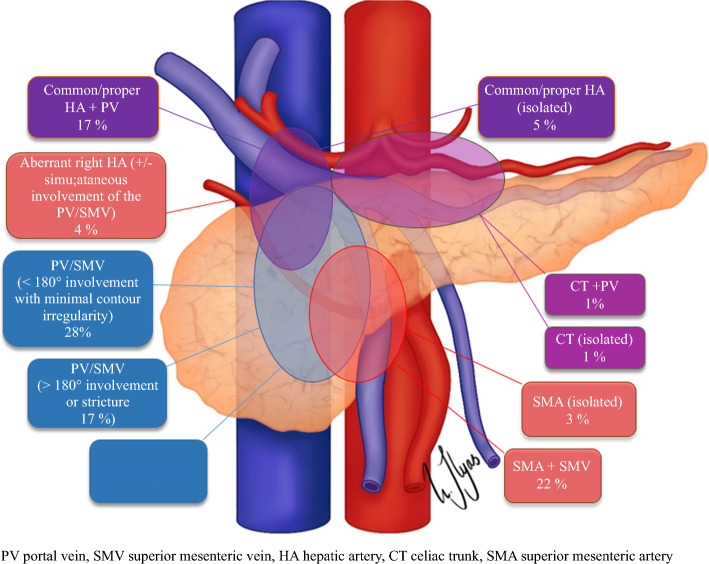
Distribution pattern of the different types of vascular involvement in the NCCN-BRPC cohort

The distribution pattern of vascular involvement of the 96 patients with NCCN-BRPC is shown in Fig. 3.

In total, 46 (47.9%) of the patients had venous involvement, 12 (12.5%) had arterial involvement, and 38 (39.6%) had combined venous and arterial involvement. Pre-chemotherapy tumor size was similar in the groups, while patients with combined venous and arterial involvement had significantly larger tumors after primary chemotherapy (*p* = 0.021; Supplementary Table 2). In the regression analysis, simultaneous arterial and venous involvement (OR 0.22, *p* = 0.038) and post-neoadjuvant CA19-9 (OR 0.99, *p* = 0.048) predicted the likelihood of resection (Table 2). Patients with either venous or arterial invasion had similar resection rates. RECIST progression and the need for chemotherapeutic switch during primary chemotherapy indicated lower resectability rates.

**Table 2:** Univariate and multivariate regression analysis of variables associated with surgical resection and overall survival in patients with NCCN-BRPC receiving chemotherapy

Baseline variables	Number	Resection				Survival			
		Univariate		Multivariate		Univariate		Multivariate	
		OR	*p*	OR	*p*	HR	*p*	HR	*p*
Tumor size < 30 mm (post-chemo)	57	1 (Ref)	NA	1 (Ref)	NA	1 (Ref)	NA		
Tumor size > 30 mm (post-chemo)	33	**0.32 (0.13–0.79)**	**0.013**	0.40 (0.10–1.61)	0.198	1.29 (0.80–2.07)	0.294		
CA19-9 < 500 kU/l (pre-chemo)	51^#^	1 (Ref)	NA			1 (Ref)	NA	1 (Ref)	NA
CA19-9 > 500 kU/l (pre-chemo)	33	0.42 (0.17–1.05)	0.064			**2.18 (1.32–3.59)**	**0.002**	**2.19 (1.24–3.87)**	**0.007**
CA19-9 post chemotherapy value*	88^#^	**0.99 (0.99–0.99)**	**0.012**	**0.99 (0.99-0.99)**	**0.048**	**1.001 (1.001–1.001)**	**0.004**^**b**^		
CA19-9 no decrease by 50 %	51^##^	1 (Ref)	NA	1 (Ref)	NA	1 (Ref)	NA		
CA19-9 decrease by 50 %	31	**2.16 (1.19-3.91)**	**0.011**	2.64 (0.89–7.80)	0.078	0.78 (0.58–1.07)	0.123		
Type of vascular invasion									
Vein only	46	1 (Ref)	NA	1 (Ref)	NA	1 (Ref)	NA		
Artery only	12	0.70 (0.19–2.52)	0.589	0.17 (0.02–1.13)	0.066	0.69 (0.34–1.44)	0.329		
Vein and artery	38	**0.32 (0.13-0.79)**	**0.014**	**0.22 (0.05-0.92)**	**0.038**	1.22 (0.76–1.97)	0.408		
Type of chemotherapy (NAT) FOLFIRINOX GnP Gemcitabine mono Other^a^	52 26 13 3	1 (Ref) 1.08 (0.42–2.77) 0.48 (0.13–1.76)	NA 0.873 0.268			1 (Ref) 1.46 (0.87–2.44) 1.07 (0.55–2.11)	NA 0.151 0.835		
Chemotherapy switch No Yes	84 12	1 (Ref) **0.08 (0.01–0.67)**	NA **0.019**	1 (Ref) **0.07 (0.01-0.74)**	NA **0.028**	1 (Ref) **3.06 (1.59-5.85)**	NA **0.001**	1.40 (0.66–2.97)	0.376
RECIST Complete/Partial response Stable disease Progression	10 62 20	1 (Ref) 1.57 (0.32–7.64) **0.05 (0–0.39)**	NA 0.733 **0.004**	1 (Ref) 2.79 (0.41–21.8) **0.08 (0-0.81)**	NA 0.387 **0.031**	1 (Ref) 1.31 (0.59–2.92) **4.22 (1.71–10.41)**	NA 0.513 **0.002**	1 (Ref) 1.09 (0.38–3.19) **4.03 (1.26–12.96)**	NA 0.863 **0.019**

Significant values are highlighted in bold

^a^Not included in analysis owing to small sample size (< 10)

^b^Not included in multivariate analysis owing to high correlation to CA19-9 > 500 at baseline

^*^Continuous variable describing OR and HR with every unit increase in CA19-9

^#^Pre-chemo CA19-9 was available in 84 cases, and post-chemo in 88 cases

^##^82 cases had CA19-9 available both pre- and post-chemo

*GnP* gemcitabine/nab-paclitaxel

Independent predictors of worse OS included baseline CA19-9 levels > 500 kU/L (HR 2.19, *p* = 0.007) and RECIST progression during neoadjuvant chemotherapy (HR 4.03, *p* = 0.019; Table 2).

**Fig. 4:**
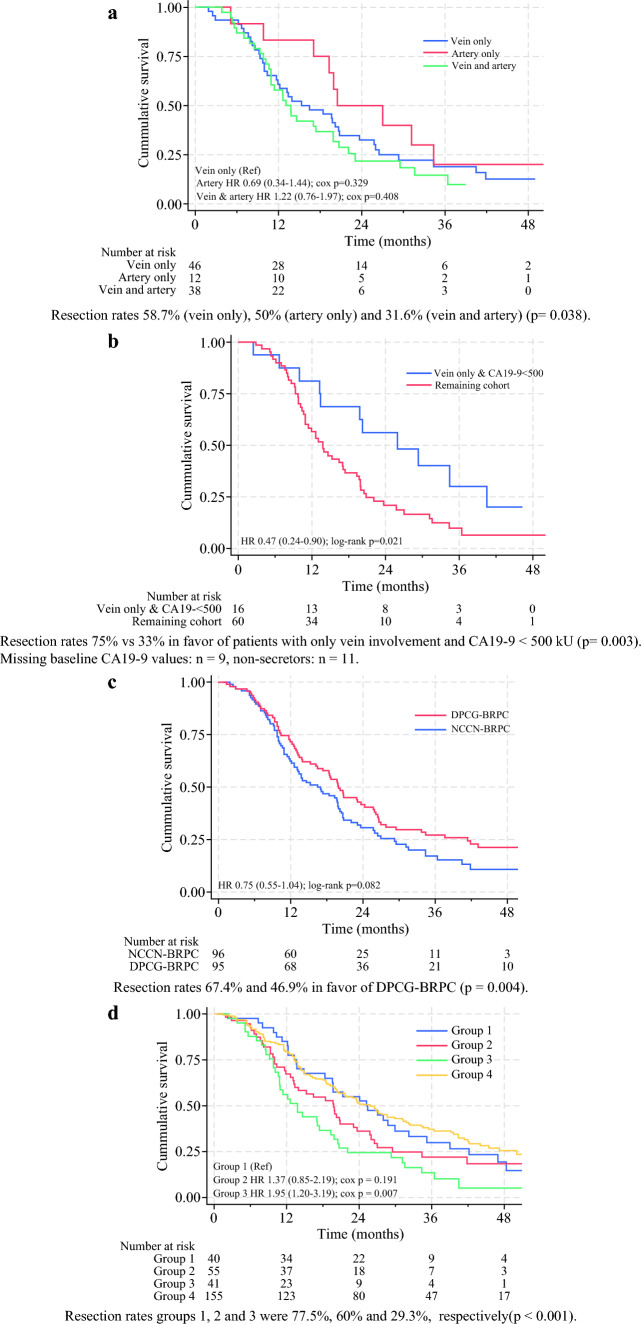

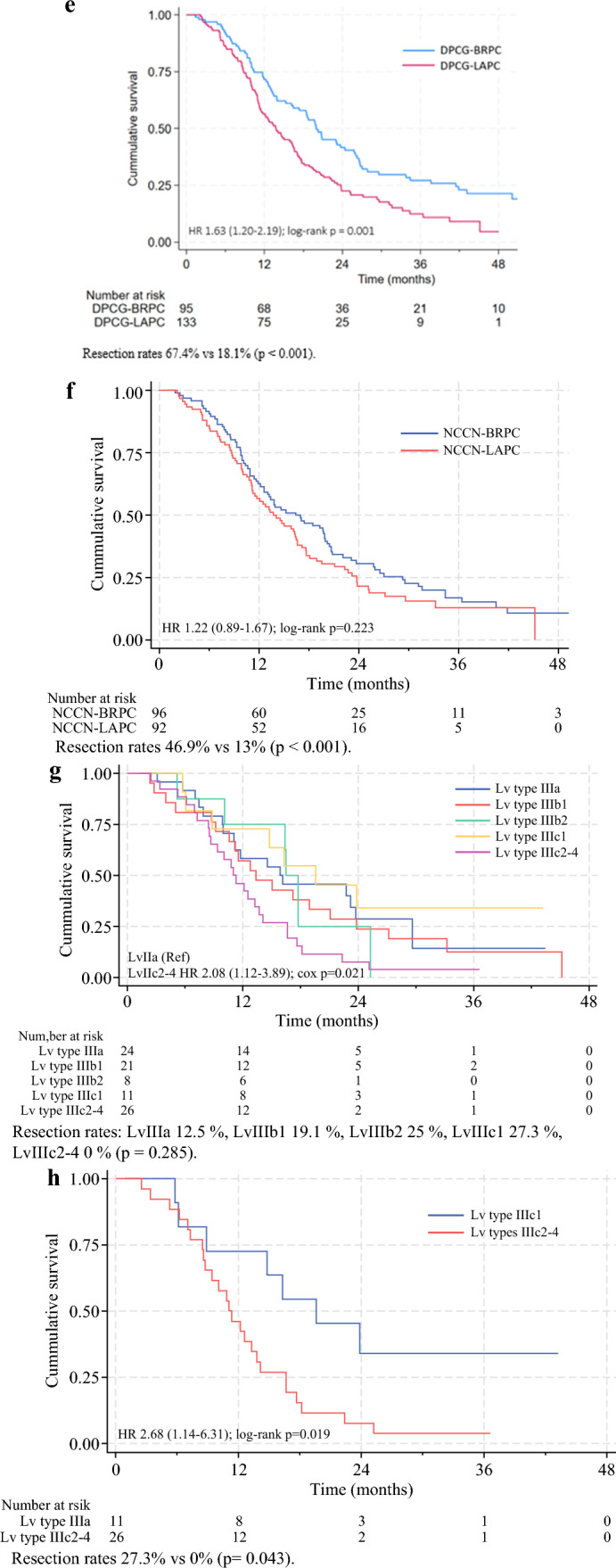
**a** Overall survival stratified by type of vascular involvement in the NCCN-BRPC cohort. **b** Overall survival in patients in the NCCN-BRPC cohort with only vein involvement and baseline CA19-9 < 500 kU/L. **c** Overall survival in patients classified as DPCG-BRPC compared with NCCN-BRPC. **d** Overall survival was compared across the three groups that were reclassified between primary resectable, BRPC, and LAPC when contrasting the DPCG and NCCN guidelines. 1—NCCN primary resectable cases reclassified as DPCG-BRPC (*n* = 40). 2—Patients with NCCN-BRPC excluding patients reclassified to DPCG-LAPC (*n* = 55). 3—Patients with NCCN-BRPC reclassified as DPCG-LAPC (*n* = 41). 4—For numerical comparison, NCCN primary resectable cases that remained primary resectable in DPCG (*n* = 155). **e** Overall survival in DPCG-BRPC and DPCG-LAPC. **f** Overall survival in NCCN-BRPC and NCCN-LAPC. **g** Overall survival in patients with NCCN-LAPC according to the Louisville types. **h** Overall survival in Lv type IIIc1 (nonreconstructable invasion of the SMV/PV without arterial involvement) and Lv types IIIc2–4 (nonreconstructable invasion of the SMV/PV with involvement of the CT, SMA, or both)

Type of vascular involvement predicted resectability, but not survival, when comparing the three groups, as shown in Table 2 and Fig. 4a. However, when creating a composite predictor of patients with any type of vein involvement (within the NCCN-BRPC definition) and a baseline CA 19-9 level < 500 kU/L (*n* = 16), we observed improved survival in addition to higher resectability rates. These patients had a resection rate of 75% versus 33.3% in the remaining BRPC cohort (*n* = 60), which in regression analysis resulted in a sixfold increase in the likelihood of resection (OR 5.99, 95% CI 1.71–20.99, *p* = 0.005) and a 50% improvement in OS (HR 0.47, 95% CI 0.24–0.90, *p* = 0.024; Fig. 4b). This difference was even more pronounced in patients with < 180˚ vein involvement with minimal vein contour irregularity (*n* = 13), showing an OR of 9.31 for resection (95% CI 1.91–45.31, *p* = 0.006) and a HR of 0.45 (95% CI 0.22–0.95, *p* = 0.037).

### Locally Advanced Pancreatic Cancer (LAPC)

None of the patients who received other chemotherapy regimens than FOLFIRINOX were explored, while 24% of those who received FOLFIRINOX were resected (*p* = 0.018, Table 3 and Fig. 2). In addition, 11% of the cohort achieved normalization of their CA19-9 values during primary chemotherapy, a group that had higher resection rates (OR 46.2, *p* = 0.013). The resection rate fell from 18 to 9% when baseline CA19-9 was > 500 kU/L, but these cohorts were underpowered, and it did not reach statistical significance (*p* = 0.096).

**Table 3:** Univariate and multivariate regression of variables associated with surgical resection and overall survival in patients with NCCN-LAPC receiving chemotherapy

Baseline variables	Number	Resection				Survival			
		Univariate		Multivariate		Univariate		Multivariate	
		OR	p	OR	P	HR	p	HR	p
Louisville classification IIIa IIIb1 IIIb2^b^ IIIc1 IIIc2-4*	24 21 8 11 26	1 (Ref) 1.63 (0.24–12.68) 2.55 (0.28–23.34) 0.22 (0–2.17)	NA 0.844 0.538 0.207			1 (Ref) 1.25 (0.65–2.44) 0.78 (0.32–1.89) **2.08 (1.12**–**3.89)**	NA 0.501 0.583 **0.021**	1 (Ref) 0.79 (0.35–1.78) 0.65 (0.23–1.79) 0.97 (0.41–2.28)	NA 0.564 0.400 0.941
Tumor size < 30 mm (post-chemo) Tumor size > 30 mm (post-chemo)	18 72	1 (Ref) **0.18 (0.055**–**0.66)**	**0.009**	**0.04 (0.01**–**0.66)**	**0.025**	1 (Ref) 1.41 (0.79–2.54)	0.247		
CA19-9 < 500 kU/l (pre-chemo) CA19-9 > 500 kU/l (pre-chemo)	45^#^ 34	1 (Ref) 0.45 (0.11–1.83)	NA 0.264			1 (Ref) 1.53 (0.93–2.52)	NA 0.096		
CA19-9 post-chemotherapy value**	77^#^	**0.99 (0.98**–**0.99)**	**0.047**	0.99 (0.98–1.00**)**	0.195	1.00 (0.99–1.00)	0.115		
CA19-9 normalization (no) CA19-9 normalization (yes)	67^#^ 10	1 (Ref) **14.76 (2.89**–**75.4)**	NA **0.001**	1 (Ref) **46.2 (2.24**–**951.9)**	NA **0.013**	1 (Ref) **0.23 (0.07**–**0.74)**	NA **0.014**	1 (Ref) 0.31 (0.09–1.07)	NA 0.064
CA19-9 no decrease by 50 % CA19-9 decrease by 50 %	49^#^ 28	1 (Ref) **4.05 (1.06**–**15.49)**	NA **0.041**^**a**^			1 (Ref) **0.45 (0.25**–**0.79)**	NA **0.006**^**a**^		
Primary chemotherapy regimen FOLFIRINOX GnP Gemcitabine mono Other^b^	51 22 14 5	1 (Ref) **0.11 (0**–**0.72)** 0.17 (0–1.18)	NA **0.017** 0.079	**0.05 (0**–**0.74)**	**0.028**	1 (Ref) 1.58 (0.91–2.75) **3.14 (1.68**–**5.87)**	NA 0.105 **<0.001**	1 (Ref) 1.68 (0.79–3.58) 1.99 (0.80–5.02)	NA 0.175 0.140
Chemotherapeutic switch No Yes	74 18	1 (Ref) 0.80 (0.16–4.02)	NA 0.786			1 (Ref) 0.89 (0.49–1.59)	NA 0.696		
RECIST Complete/partial response Stable disease Progression	10 59 23	1 (Ref) 0.16 (0.02–1.06) **0.04 (0**–**0.40)**	NA 0.059 **0.004**	1 (Ref) 0.56 (0.03–7.10) 0.84 (0–11.55)	NA 0.966 0.902	1 (Ref) 1.93 (0.76–4.91) **8.88 (3.13**–**24.9)**	NA 0.167 **<0.001**	1 (Ref) 1.71 (0.47–6.18) **8.02 (2.02**–**31.9)**	NA 0.416 **0.003**

Significant values are highlighted in bold

^*^Owing to small sample sizes and similarities within these groups, LvIIIc2–4 were collected into a single group

^**^Continuous variable describing OR and HR with every unit increase in CA19-9

^a^Not included in multivariate analysis owing to high correlation to CA19-9 normalization

^b^Not included in analysis owing to small sample size (< 10)

^#^Pre-chemo CA19-9 was available in 79 cases, and post-chemo in 77 cases

*GnP* gemcitabine/nab-paclitaxel

**Fig. 5:**
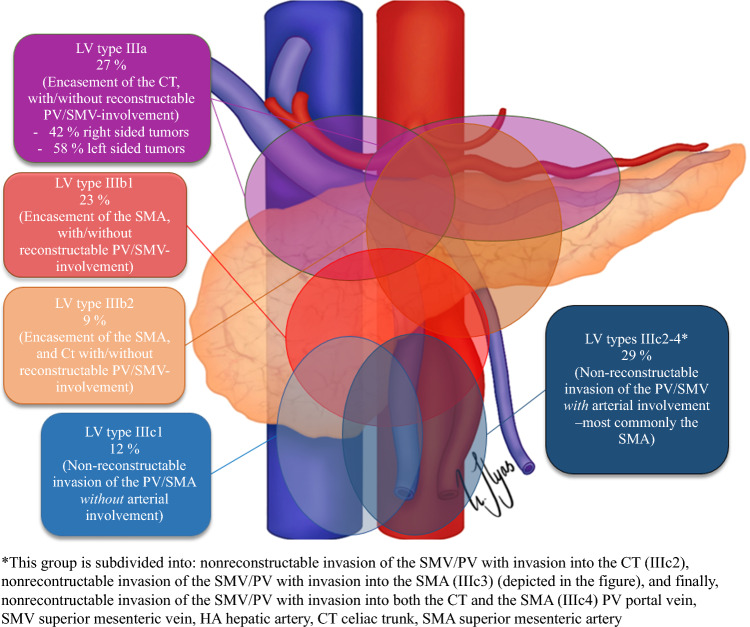
Distribution pattern of the different stages of vascular involvement according to the Louisville classification system in the NCCN-LAPC cohort

The Louisville staging system (shown in Fig. 5) predicted worse survival outcomes for a subset of patients with LAPC, specifically those with nonreconstructable invasion of the portal vein/superior mesenteric vein (PV/SMV) combined with arterial involvement (Lv types IIIc2–4; HR 2.08, *p* = 0.021; Fig. 4g). As previously demonstrated from this cohort, LAPC differs from BRPC in that the former is more frequently located in the body and tail of the pancreas (28% versus 9%, *p <* 0.001).[4] However, tumors considered locally advanced due to unresectable vein involvement, with or without concurrent arterial involvement (Lv type IIIc1–c4), predominantly occurred in the head of the pancreas (90%) and tended to extend below the level of the duodenum into the root of the mesentery (Fig. 5). We observed a significant distinction between tumors deemed unresectable owing to vein involvement alone (Lv IIIc1) and those that had unresectable vein involvement with the addition of arterial involvement (Lv IIIc2–4), most commonly the SMA. The additional involvement of the artery reduced the probability of resection (27.3% versus 0%, OR 0.09, *p* = 0.043), resulting in shorter overall survival (median 11 versus 20 months, HR 2.68, *p* = 0.024; Fig. 4h).

**Fig. 6:**
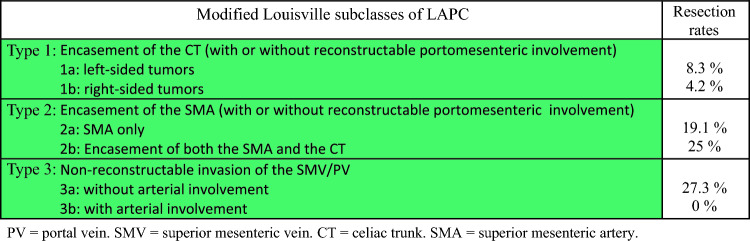
Modified Louisville classification of NCCN-LAPC and the probability of surgical resection in patients eligible for primary chemotherapy

The other two groups that had higher resection rates were Lv types IIIb1 and b2 (19.1% and 25%, respectively). Together with the Lv type IIIc1 tumors, these patients had a significant higher resection rate than the remaining LAPC cohort (22.5% versus 5.7%, *p* = 0.023). No difference in resection rates were found when isolating the tumors with encasement of the celiac trunk (CT; Lv type IIIa) and subdividing these into left- and right-sided tumors. Figure 6 shows a modified version of the Louisville types and the rates at which they reached resection.

### The Dutch Classification System (DPCG)

In total, 40 out of 195 patients (20.5%), initially classified as having primary resectable tumors according to the NCCN definition, were reclassified to DPCG-BRPC. In addition, 41 out of 96 patients (42.7%), initially classified as NCCN-BRPC, were reclassified to DPCG-LAPC. Figure 4d shows the survival curves for the three groups that shifted between the categories.

Comparing the DPCG-BRPC and NCCN-BRPC cohorts, patients in the Dutch group had a higher likelihood of undergoing resection (67.4% versus 46.9%, *p* = 0.004), with an OR of 2.34 (95% CI 1.30–4.21, *p* = 0.005). Furthermore, the need for vascular resection was lower among patients with DPCG-BRPC (36.9% versus 57.8%, *p* = 0.031; Supplementary Table 4). Figure 4c shows the difference in trends with regards to survival.

For LAPC, there were no significant differences in resection rates or survival when comparing DPCG with NCCN, with resection rates of 18.1% versus 13% (*p* = 0.314), and a HR of 1.00 (95% CI 0.75–1.34, *p* = 0.998).

In the DPCG cohort, the resection rate was significantly lower for LAPC compared with BRPC (18.1% versus 67.4%; OR 0.11, 95% CI 0.06–0.20, *p <* 0.001), similar to the findings using the NCCN system (13% versus 46.9%; OR 0.17, 95% CI 0.08–0.35, *p <* 0.001). The DPCG system successfully predicted worse survival for LAPC compared with BRPC (HR 1.63, 95% CI 1.20–2.20, *p* = 0.002), a prediction that the NCCN system failed to make (HR 1.22, 95% CI 0.89–1.67, *p* = 0.224; Fig. 4e and f). Consequently, the DPCG system proved to be effective at predicting both resectability and survival when comparing BRPC to LAPC, whereas the NCCN system only predicted resectability.

The Dutch system also demonstrated superior predictive ability for survival in patients with baseline CA19-9 levels > 500 kU/L. As presented in Tables 2 and 3, this parameter predicted survival in NCCN-BRPC (*p* = 0.007) but did not significantly predict survival in NCCN-LAPC (*p* = 0.096). However, under the Dutch system, CA19-9 > 500 kU/L was a significant predictor of survival for both BRPC and LAPC, with HR 2.84 (95% CI 1.38–5.82, *p* = 0.004) and HR 1.54 (95% CI 1.03–2.32, *p* = 0.037), respectively.

## Discussion

This subgroup analysis of the NORPACT-2 study cohort demonstrated a significant distribution difference in BRPC and LAPC according to the DPCG and the NCCN classification systems. In fact, 20.5% and 42.7% of patients initially classified as primary and borderline resectable according to NCCN where moved to BRPC and LAPC according to DPCG. Patients classified as DPCG-BRPC had significantly higher resection rates and lower likelihood of needing vascular resection, when compared with NCCN-BRPC. The subgroup classified as BRPC by NCCN but LAPC by DPCG had particularly low resection rates and poor survival outcomes. These findings highlight the need for one unified system, enabling results from trials to be easily compared.

Furthermore, this study demonstrates that subclassifying tumor anatomy, on the basis of varying degrees of vascular invasion, along with tumor biology, on the basis of baseline CA19-9 and CA19-9 dynamics, can effectively predict resectability rates and survival outcomes for patients with BRPC and LAPC. This illustrates that these groups indeed represent a heterogeneous cohort with varying survival and resection rates.

BRPC encompasses a wide range of patients, from those with minimal vein involvement to those with involvement of both the veins and arteries. In this study, the NCCN criteria did not predict survival for BRPC cohort as a whole. However, it was possible to predict varying survival outcomes within subsets of this heterogeneous group. In this audit, BRPC with either venous or arterial involvement resulted in similar outcomes. However, when two vessels were affected, the OR for achieving successful resection dropped to 0.22. Patients with the composite predictor of only vein involvement and baseline CA19-9 < 500 kU/L demonstrated significantly higher resectability rates compared with the rest of the BRPC-cohort (75% versus 33%) and a 50% improvement in OS. This resection rate is closer to what one will find in primary resectable tumors, perhaps indicating that this subset of BRPC may not benefit from prolonged neoadjuvant therapy. As such, these findings illustrate that, within the existing NCCN classification system, there is further room for stratification, which could influence and improve neoadjuvant strategies. Excluding patients with high baseline CA19-9 levels from this group is reasonable, as levels > 500 kU/L are unlikely to be explained by vein involvement alone and often indicate occult metastatic disease. In such cases, a prolonged “test of time” approach with neoadjuvant therapy is recommended.

When applying the Dutch classification system to our study population, 20.5% of patients with NCCN-defined primary resectable tumors were reclassified to DPCG-BRPC, while 42.7% of NCCN-BRPC were reclassified to DPCG-LAPC. The shifts from NCCN primary resectable and BRPC to DPCG-BRPC and LAPC led to significant differences in both resectability rates and survival outcomes, highlighting the challenges of comparing studies that apply different classification systems. In particular, the group that was reclassified from NCCN-BRPC to DPCG-LAPC had worse outcomes regarding both resectability and survival (Fig. 4d). This highlights a subset of patients that is likely to affect outcomes in trials examining NCCN-BRPC, which make comparisons with DPCG-BRPC difficult to interpret. This subset of patients also likely influenced our inability to detect a survival difference between BRPC and LAPC using the NCCN criteria, whereas the DPCG system allowed for such differentiation (Fig. 4e and f). The PREOPANC-1 study randomized patients with DPCG primary resectable tumors and DPCG-BRPC to receive either neoadjuvant chemoradiotherapy with gemcitabine or upfront surgery followed by adjuvant gemcitabine. The study demonstrated reduced rates of pancreatic fistulas and improved survival, particularly for the BRPC group in the neoadjuvant cohort.^15^ The PREOPANC-2 and PREOPANC-3 studies, likewise, define primary resectable PC and BRPC according the Dutch criteria.^13,16^ According to the Dutch classification, these would represent BRPC tumors with minor vascular involvement, a group that has shown favorable outcomes in our study. Consequently, interpreting the results of these studies within the NCCN system presents considerable challenges.

One may argue that the term BRPC should be limited to patients with technically resectable tumors that have a high likelihood of requiring vascular resections, while LAPC should denote unresectability. While the Dutch classification system was better than NCCN in predicting survival outcomes, it fell short in distinguishing between primary resectable and BRPC, as it categorizes a disproportionately high number of the former as BRPC and inaccurately predicts the necessity for vascular resections, with only 37% of cases actually requiring such procedures. Furthermore, our research has previously demonstrated that extending procedures to include venous resections should be based on clear radiologic or macroscopic signs of invasion. Extended procedures as a means of achieving more R0 resections rarely achieve their goal.^17^

By applying the Louisville classification system ^8^ to our LAPC cohort, we identified distinct patient subsets with varying levels of resectability and survival outcomes. At one end of the spectrum were patients with nonreconstructable vein invasion but no arterial involvement, while at the other were those with nonreconstructable vein invasion and arterial involvement. Between these extremes, patients with tumors encasing either the SMA alone or both the SMA and the CT demonstrated higher resection rates. We did not observe a difference between tumors encasing both the CT and the gastroduodenal artery (typically right-sided tumors) and those encasing only the CT (typically left-sided tumors). This finding contrasts with a report from Johns Hopkins, which found a higher resection rate for the latter group.^9^ Although our data did not reveal a distinction, we believe there is a clinically important difference between these two tumor types, which the Louisville system fails to address. We also suggest that the Louisville types IIIc2–4 should be consolidated, given their similar clinical outcomes and the rarity of nonreconstructable vein invasion involving only the CT without SMA involvement. While the Louisville classification is relatively straightforward, we propose a modified version in Fig. 6, which offers greater clarity and has improved clinical applicability. This modification needs to be evaluated in a larger scale study.

In our study, the only “truly unresectable” tumors where those that caused a nonreconstructable vein invasion with simultaneous arterial involvement. The Johns Hopkins classification of LAPC^9^ similarly identified these tumors, together with those encasing both the CT and the gastroduodenal artery, as *LAPC-3*, or highly unlikely to undergo surgery. At the other end of the spectrum, they described *LAPC-1* as tumors with greater likelihood of undergoing resection, and in this category they included tumors that encased the common hepatic artery, those encasing the CT without involvement of the gastroduodenal artery, and tumors with isolated encasement of the SMA. Johns Hopkins reported a 63% exploration rate and a 49% resection rate for LAPC-1 tumors. By comparison, for our patient groups more likely to be operated (Lv types IIIb1, b2, and c1), we observed an exploration rate of 33% and a resection rate of 23%. This discrepancy, i.e., the narrow window between explored and resected patients in our cohort, suggests that our approach to explore LAPC may be overly restrictive. Indeed, the goal in managing LAPC should be to identify subgroups where extending surgical boundaries is justified, even at the risk of “negative” laparotomies. However, Johns Hopkins’ inclusion of tumors with common hepatic artery encasement in their LAPC category may be questioned, since these are classified as BRPC in the NCCN classification if the tumor is without extension to the CT or hepatic artery bifurcation, and a safe and complete arterial resection and reconstruction can be performed. This misclassification is likely a contributing factor to the unexpectedly high resection rate reported in their study.

A recent nationwide Norwegian study highlighted significant improvements in survival rates over the past 15 years following resection for pancreatic cancer, contrasting sharply with the lack of progress in patients who received only palliative chemotherapy, during the same period that modern combination chemotherapy regimens have been introduced.^18^ This underscores the limited efficacy of chemotherapy alone for this disease. Our recent findings have emphasized the importance of getting patients with BRPC/LAPC to surgery, as their survival rates then match those of patients with primarily resectable pancreatic cancer who undergo surgery.^4^ The best outcomes for patients with BRPC or LAPC are seen in those who undergo successful surgical resection after primary chemotherapy, together with adjuvant chemotherapy, making the decision to pursue surgery crucial.

We have emphasized the critical need for continuous assessment of neoadjuvant chemotherapy strategies in patients with BRPC, particularly as some patients, termed “nonresponders,”^19^ do not benefit from first-line treatment with FOLFIRINOX. In our study, patients who required a switch in chemotherapy after four cycles experienced a significant reduction in the likelihood of resection and a threefold increase in mortality. Progression during neoadjuvant therapy emerged as a strong predictor of both resectability and survival. Therefore, the routine use of eight cycles of first-line chemotherapy in patients with BRPC administered in some trials without a careful, periodic re-evaluation and potential change in treatment strategy may be questioned. Our findings indicate that while RECIST stable disease reflects no deterioration, progression under chemotherapy indicates the need for an immediate change in treatment strategy.

Several studies have investigated the effects of various neoadjuvant treatment strategies for BRPC.^20–23^ It is essential to determine whether these are based on an intention-to-treat analysis from the time of radiologic diagnosis or if they report outcomes for patients who have already undergone invasive preoperative diagnostics and some level of neoadjuvant therapy. The latter represents a more selective cohort, potentially with more favorable outcomes. Combining data from different registries without considering these differences in an effort to better understand neoadjuvant treatment strategies often raises more questions than it answers and should be avoided.^24^

The strengths of this study include the use of a population-based, nonselective cohort treated within a universal healthcare system. Consecutive patients were prospectively enrolled following a protocol-based approach guided by the intention-to-treat principle. Moreover, we strictly adhered to the widely accepted NCCN criteria for defining BRPC and LAPC. This study has some limitations. First, the small sample size of 228 patients, with only 45 BRPC and 12 LAPC cases undergoing resection, limited the statistical power, causing some results to lack significance and some subgroup analyses to be very small. Second, while interpretation of radiology, endoscopic ultrasound (EUS) diagnostics, and pancreatic cancer surgeries are centralized at a single center in our region, neoadjuvant therapy is administered across ten different institutions. Although all patients are initially evaluated and treatment sequences are determined at our center, including at various stages of response evaluation, local oncological institutions decide on the specific chemotherapy regimens, leading to potential variations in treatment strategies. Lastly, although this is a single-center study, which may limit external validity, the catchment area of 3.1 million people ensures substantial surgical experience and a dedicated team within a high-volume setting.

## Supplementary Information

Below is the link to the electronic supplementary material.

Supplementary file1 (DOCX 42 KB)
