# Supplementary material for: Anatomical Classification and Staging Systems of Borderline Resectable and Locally Advanced Pancreatic Cancer—A Subgroup Analysis of the NORPACT-2 Trial
Source: Ann Surg Oncol. 2025 Jun 3;32(12):8856–69. doi: 10.1245/s10434-025-17527-y (PMC12534251; doi:10.1245/s10434-025-17527-y)
Supplement: Supplementary file 1 — Supplementary file1 (DOCX 42 KB) [file 10434_2025_17527_MOESM1_ESM.docx]

SUPPLEMENTARY TABLES

**Supplementary Table 1**

Baseline and treatment characteristics for patients with NCCN-primary resectable pancreatic cancer reclassified to DPCG-BRPC (n=40, of a total of 198 patients)

|  | NCCN-primary reclassified to DPCG-BRPC |
| --- | --- |
|  | N=40 |
| Age, mean (95 % CI) | 67 (63.3-70.5) |
| Sex ratio (male:female) | 22 : 18 |
| Body mass index, mean (95 % CI) | 26.7 (24.2-27.1) |
| Performance status (ECOG), n (%) |  |
| 0 | 22 (57.9) |
| 1 | 11 (28.9) |
| >1 | 5 (13.2) |
| Charlson comorbidity index, n (%) |  |
| 0 | 23 (57.5) |
| 1 | 12 (30) |
| >1 | 5 (12.5) |
| Biliary drainage, n (%) | 25 (65.8) |
| CA19-9 at baseline (kU/L), median (IQR) | 158 (44-814) |
| Tumor location, n (%) |  |
| Head/uncinate process | 32 (80) |
| Body/tail | 8 (20) |
| Tumor size pre chemotherapy (mm), mean (95 % CI) | 27 (24.4-30) |
| Upfront surgery, n (%) | 34 (85) |
| Neoadjuvant chemotherapy, n (%)* | 6 (15) |
| Surgical resection, n (%) | 31 (77.5) |
| Types of procedures, n (%) |  |
| Pancreatoduodenectomy | 27 (87.1) |
| Distal pancreatectomy | 5 (12.9) |
| Total pancreatectomy | 0 |
| Concomitant vascular resection, n (%) | 4 (14.8) |
| Postoperative complications ≥ Clavien grade 3, n (%) | 4 (14.8) |
| Adjuvant chemotherapy, n (%) | 25 (80.1) |
| Overall survival (months), median (IQR) | 23.7 (12.4-43.9) |

*All 6 patients received FOLFIRINOX.

**Supplementary Table 2**

Baseline and treatment characteristics of NCCN-BRPC patients receiving chemotherapy, stratified by type of vascular invasion

|  | NCCN BRPC | | | |  |
| --- | --- | --- | --- | --- | --- |
|  | Overall  n=96 | Vein  n=46 | Artery  n=12 | Vein + Artery  n=38 | p-value |
| Age, mean (95 % CI) | 69 (67.1-70.7) | 68 (65.5-71.1) | 71 (66.8.2-74.9) | 70 (67.2-72.2) | 0.588 |
| Sex ratio (male:female) | 47 : 49 | 22 : 24 | 8 : 4 | 17 : 21 | 0.406 |
| Body mass index, mean (95 % CI) | 24 (22.9-24.6) | 24 (22.4-24.9) | 24 (21.4-25.9) | 24 (22.8-25.6) | 0.829 |
| Performance status (ECOG), n (%) |  |  |  |  | **0.045** |
| 0 | 52 (54.2) | 20 (43.5) | 8 (66.7) | 24 (63.2) |  |
| 1 | 37 (38.5) | 23 (50) | 3 (25.0) | 11 (28.9) |  |
| >1 | 7 (7.3) | 3 (6.5) | 1 (8.3) | 3 (7.9) |  |
| Charlson comorbidity index, n (%) |  |  |  |  | 0.423 |
| 0 | 48 (50) | 26 (56.5) | 6 (50) | 16 (42.1) |  |
| 1 | 33 (34.4) | 16 (34.8) | 4 (33.3) | 13 (34.2) |  |
| >1 | 15 (15.6) | 4 (8.7) | 2 (16.7) | 9 (23.7) |  |
| Biliary drainage, n (%) | 63 (65.6) | 31 (67.4) | 9 (75) | 23 (60.5) | 0.616 |
| CA19-9 at baseline (kU/L), median (IQR) | 323 (72-953) | 352 (113-998) | 254 (140-931) | 256 (47-901) | 0.624 |
| Tumor location, n (%) |  |  |  |  | 0.252 |
| Head/uncinate process | 87 (90.6) | 44 (95.7) | 10 (83.3) | 33 (86.8) |  |
| Body/tail | 9 (9.4) | 2 (4.3) | 2 (16.7) | 5 (13.2) |  |
| Tumor size pre chemotherapy (mm), mean (95 % CI) | 30 (25.5-36.5) | 30 (27.5-32.3) | 29 (25.8-32.7) | 33 (29.8-36) | 0.207 |
| Chemotherapy regimen, n (%) |  |  |  |  | 0.504 |
| FOLFIRINOX | 52 (54.2) | 22 (47.8) | 7 (58.3) | 23 (60.5) |  |
| Gemcitabine/Nab-paclitaxel | 26 (27.1) | 16 (34.8) | 2 (16.7) | 8 (21.1) |  |
| Gemcitabine | 13 (13.5) | 5 (10.9) | 3 (25) | 5 13.2) |  |
| Other regimen | 3 (3.1) | 1 (2.1) | 0 | 2 5.3) |  |
| Upfront surgery | 2 (2.1) | 2 (4.4) | 0 | 0 |  |
| Chemotherapeutic switch, n (%) | 12 (12.5) | 4 (8.7) | 0 | 8 (21.1) | 0.088 |
| CA19-9 post chemotherapy (kU/L), median (IQR) | 166 (56-541) | 214 (86-599.5) | 214 (43-315) | 147 (39-612) | 0.764 |
| Tumor size post chemotherapy (mm) , mean (95 % CI) | 27 (21-33) | 25 (23.7-28.8) | 26 (20.8-31.1) | 33 (28.4-36.8) | **0.021** |
| Response Evaluation Criteria In Solid Tumours at restaging, n (%) |  |  |  |  | 0.479 |
| Complete/Partial response | 10 (10.9) | 4 (9.5) | 3 (25) | 3 (7.9) |  |
| Stable disease | 62 (67.4) | 30 (71.4) | 7 (58.3) | 25 (65.8) |  |
| Progressive disease | 20 (21.7) | 8 (19.1) | 2 (16.7) | 10 (26.3) |  |
| Surgical resection, n (%) | 45 (45.9) | 27 (58.7) | 6 (50) | 12 (31.6) | **0.045** |
| Types of procedures, n (%) |  |  |  |  | 0.356 |
| Pancreatoduodenectomy | 42 (93.3) | 26 (96.3) | 5 (83.3) | 11 (91.7) |  |
| Distal pancreatectomy | 2 (4.5) | 0 | 1 (16.7) | 1 (8.3) |  |
| Total pancreatectomy | 1 (2.2) | 1 (3.7) | 0 | 0 |  |
| Concomitant vascular resection, n (%) | 26 (57.8) | 18 (66.7) | 1 (16.7) | 7 (58.3) | 0.081 |
| Postoperative complications ≥ Clavien grade 3, n (%) | 9 (20) | 6 (22.2) | 1 (16.7) | 2 (16.7) | 0.901 |
| Adjuvant chemotherapy, n (%) | 34 (75.6) | 20 (74.1) | 5 (83.3) | 9 (75) | 0.891 |
| Overall survival (months), median (IQR) | 16.8 (9.8-25.2) | 15.9 (9.4-26) | 20.7 (18.2-32.8) | 13.5 (9.8-20.7) | 0.169 |

**Supplementary Table 3**

Baseline and treatment characteristics of NCCN-LAPC patients receiving chemotherapy, stratified by the Louisville classification system

|  | Overall (n=92*) | NCCN LAPC (Louisville types) | | | | |  |  | p-value |
| --- | --- | --- | --- | --- | --- | --- | --- | --- | --- |
|  |  | IIIa | IIIb1 | IIIb2 | IIIc1 | IIIc2 | IIIc3 | IIIc4 |  |
|  |  | N=24 | N=21 | N=8 | N=11 | N=2 | N=17 | N=7 |  |
| Age, means (95 % CI) | 66 (64.3-68.29) | 64 (60.6-67.5) | 66 (61.4-70.5) | 66 (60.6-71) | 64 (55.8-71.4) | 74 (66.1-81.9) | 70 (66.3-72.9) | 67 (60-72.3) | 0.456 |
| Sex ratio (male:female) | 51 : 41 | 13:11 | 10:11 | 5:3 | 9:2 | 2:0 | 6:11 | 4:3 | 0.228 |
| Body mass index, mean (95 % CI) | 24 (23.5-25.4) | 26 (23.1-27.9) | 24 (22.6-26.4) | 24 (20.9-27.3) | 24 (22.2-26.2) | 23 (21-24.9) | 24 (21.8-25.9) | 23 (19.3-27.2) | 0.905 |
| Performance status (ECOG), n (%) |  |  |  |  |  |  |  |  | 0.783 |
| 0 | 58 (63) | 16 (66.7) | 11 (52.4) | 4 (50) | 7 (63.6) | 2 (100) | 11 (64.7) | 5 (71.4) |  |
| 1 | 27 (29.4) | 8 (33.3) | 7 (33.3) | 3 (37.5) | 4 (36.4) | 0 | 4 (23.5) | 1 (14.3) |  |
| >1 | 7 (7.6) | 0 | 3 (14.3) | 1 (12.5) | 0 | 0 | 2 (11.8) | 1 (14.3) |  |
| Charlson comorbidity index, n (%) |  |  |  |  |  |  |  |  | 0.728 |
| 0 | 47 (51.1) | 11 (45.9) | 14 (66.7) | 3 (37.5) | 5 (45.5) | 1 (50) | 8 (47.1) | 4 (57.1) |  |
| 1 | 28 (38.4) | 9 (37.5) | 5 (23.8) | 2 (25) | 4 (36.4) | 1 (50) | 5 (29.4) | 1 (14.3) |  |
| >1 | 17 (18.5) | 4 (16.6) | 2 (9.5) | 3 (37.5) | 2 (18.1) | 0 | 4 (23.5) | 2 (28.6) |  |
| Biliary drainage, n (%) | 37 (40.2) | 5 (20.8) | 12 (57.1) | 0 | 7 (63.6) | 1 (50) | 8 (47.1) | 3 (42.9) | **0.024** |
| CA19-9 at baseline (kU/L), median (IQR) | 345 (75-1073) | 269 (119-1001) | 401 (59-942) | 384 (70.5-796) | 81 (58-825) | 3540 | 1055 (40-2468) | 243 (89-2662) | 0.702 |
| Tumor location, n (%) |  |  |  |  |  |  |  |  | **< 0.001** |
| Head/uncinate process | 66 (71.7) | 10 (41.7) | 19 (90.5) | 2 (25) | 10 (90.9) | 2 (100) | 15 (88.2) | 7 (100) |  |
| Body/tail | 26 (28.3) | 14 (58.3) | 2 (9.5) | 6 (75) | 1 (9.1) | 0 | 2 (11.8) | 0 |  |
| Tumor size pre chemotherapy (mm), mean (95 % CI) | 44 (40.5-46.7) | 42 (37.3-46.3) | 37 (33.3-42.9) | 51 (39.3-62.2) | 39 (34.3-44.4) | 80 (40.3-119.7) | 45 (41.4-49.5) | 50 (32.7-67.3) | **0.010** |
| Chemotherapy regimen, n (%) |  |  |  |  |  |  |  |  | **0.012** |
| FOLFIRINOX | 51 (55.4) | 19 (79.2) | 13 (61.9) | 6 (75) | 6 (54.5) | 0 | 3 (17.7) | 3 (42.9) |  |
| Gemcitabine/Nab-paclitaxel | 22 (23.9) | 2 (8.3) | 3 (14.3) | 2 (25) | 4 (35.4) | 0 | 7 (41.1) | 4 (57.1) |  |
| Gemcitabine | 14 (15.2) | 3 (12.5) | 4 (19.1) | 0 | 1 (9.1) | 2 (100) | 3 (17.7) | 0 |  |
| Other regimen | 5 (5.4) | 0 | 1 (4.8) | 0 | 0 | 0 | 4 (23.5) | 0 |  |
| Upfront surgery | 0 | 0 | 0 | 0 | 0 | 0 | 0 | 0 |  |
| Chemotherapeutic switch, n (%) | 18 (19.6) | 8 (33.3) | 2 (9.5) | 2 (25) | 2 (18.2) | 0 | 3 (17.7) | 1 (14.3) | 0.549 |
| CA19-9 post chemotherapy (kU/L), median (IQR) | 222 (63-641) | 241 (63-569) | 194 (59-1874) | 168 (68-351) | 64 (19-151) | 890 | 558 (228-854) | 249 (72-2256) | 0.265 |
| Tumor size post chemotherapy (mm), mean (95 % CI) | 44 (39.9-47.3) | 43 (37.2-48.2) | 38 (30.2-45.1) | 42 (28.5-55.2) | 38 (28.9-48) | 72 (164-127.6) | 48 (41.6-54.3) | 49.6 (31.2-68) | 0.084 |
| Response Evaluation Criteria In Solid Tumours at restaging, n (%) |  |  |  |  |  |  |  |  | 0.416 |
| Complete/Partial response | 10 (10.8) | 1 (4.2) | 3 (14.3) | 2 (25) | 2 (18.2) | 0 | 0 | 0 |  |
| Stable disease | 59 (64.2) | 16 (66.7) | 10 (47.6) | 5 (62.5) | 8 (72.7) | 2 (100) | 13 (76.5) | 5 (71.4) |  |
| Progressive disease | 23 (25) | 7 (29.1) | 8 (38.1) | 1 (12.5) | 1 (9.1) | 0 | 4 (23.5) | 2 (28.6) |  |
| Surgical resection, n (%) | 12 (13) | 3 (12.5) | 4 (19.1) | 2 (25) | 3 (27.3) | 0 | 0 | 0 | 0.285 |
| Types of procedures, n (%) |  |  |  |  |  |  |  |  | 0.137 |
| Pancreatoduodenectomy | 7 (58.3) | 1 (33.3) | 4 (100) | 0 | 2 (66.7) | 0 | 0 | 0 |  |
| Distal pancreatectomy | 1 (8.3) | 1 (33.3) | 0 | 0 | 0 | 0 | 0 | 0 |  |
| Total pancreatectomy | 4 (33.4) | 1 (33.3) | 0 | 2 (100) | 1 (33.3) | 0 | 0 | 0 |  |
| Concomitant vascular resection, n (%) | 8 (66.7) | 2 (66.7) | 2 (50) | 2 (100) | 2 (66.7) | NA | NA | NA | 0.682 |
| Postoperative complications ≥ Clavien grade 3, n (%) | 6 (50) | 2 (66.7) | 2 (50) | 1 (50) | 1 (33.3) | NA | NA | NA | 1.000 |
| Adjuvant chemotherapy, n (%) | 3 (25) | 1 (33.3) | 2 (50) | 0 | 0 | NA | NA | NA | 0.375 |
| Overall survival, median (IQR) | 13.9 (8.7-22.5) | 16.8 (10-23.5) | 13.4 (9.2-23.8) | 17.1 (13.3-20.7) | 19.6 (8.8-28.8) | 8.3 (3.4-13.3) | 11 (8.5-16.6) | 11.3 (8.4-16.6) | 0.419 |

*****Two patients did not fit into the Louisville classification system (both had non-resectable tumors that encased the aorta).

**Supplementary Table 4**

Baseline and treatment characteristics for patients with BRPC or LAPC, as defined by the NCCN and the DPCG systems

|  | NCCN BRPC | DPCG BRPC | p-value | NCCN LAPC | DPCG LAPC | p-value |
| --- | --- | --- | --- | --- | --- | --- |
|  | N=96 | N=95 |  | N=92 | N=133 |  |
| Age, mean (95 % CI) | 69 (67.4-70.9) | 68 (65.5-69.7) | 0.259 | 66 (64.3-68.2) | 68 (66-69.1) | 0.294 |
| Sex ratio (male:female) | 47 : 49 | 48 : 47 | 0.828 | 51 : 41 | 72 : 61 | 0.847 |
| Body mass index, mean (95 % CI) | 24 (23-24.7) | 24 (23.2-24.9) | 0.787 | 24 (23.5-25.4) | 25 (23.9-25.5) | 0.692 |
| Performance status (ECOG), n (%) |  |  | 0.706 |  |  | 0.872 |
| 0 | 52 (54.2) | 48 (51.6) |  | 58 (63) | 84 (63.2) |  |
| 1 | 37 (38.5) | 37 (38.5) |  | 27 (29.4) | 38 (28.6) |  |
| >1 | 7 (7.3) | 8 (8.6) |  | 7 (7.6) | 11 (8.2) |  |
| Charlson comorbidity index, n (%) |  |  | 0.258 |  |  | 0.962 |
| 0 | 48 (50) | 52 (54.7) |  | 47 (51.1) | 66 (46.6) |  |
| 1 | 33 (34.4) | 29 (30.5) |  | 28 (30.4) | 44 (33.1) |  |
| >1 | 15 (15.6) | 14 (14.7) |  | 17 (18.5) | 23 (17.3) |  |
| Biliary drainage, n (%) | 63 (65.6) | 58 (62.4) | 0.641 | 37 (40.2) | 67 (50.4) | 0.133 |
| CA19-9 at baseline (kU/L), median (IQR) | 323 (72-953) | 197 (50-860) | 0.375 | 345 (75-1073) | 346 (83-1073) | 0.854 |
| Tumor location, n (%) |  |  | 0.324 |  |  | 0.331 |
| Head/uncinate process | 87 (90.6) | 82 (86.3) |  | 66 (71.7) | 103 (77.4) |  |
| Body/tail | 9 (9.4) | 13 (13.7) |  | 26 (28.3) | 30 (22.6) |  |
| Tumor size pre chemotherapy (mm), mean (95 % CI) | 31 (29.3-32.7) | 29 (27.1-30.5) | 0.074 | 44 (40.5-46.7) | 40 (37.7-42.6) | 0.084 |
| Chemotherapy regimen, n (%) |  |  | 0.947 |  |  | 1.000 |
| FOLFIRINOX | 52 (54.2) | 35 (57.4) |  | 51 (55.4) | 74 (55.6) |  |
| Gemcitabine/Nab-paclitaxel | 26 (27.1) | 16 (26.2) |  | 22 (23.9) | 32 (24.1) |  |
| Gemcitabine | 13 (13.5) | 7 (11.5) |  | 14 (15.2) | 20 (15) |  |
| Other regimen | 3 (3.1) | 1 (1.6) |  | 5 (5.4) | 7 (5.3) |  |
| Upfront surgery | 2 (2.1) | 2 (3.3) |  | 0 | 0 |  |
| Chemotherapeutic switch, n (%) | 12 (12.5) | 3 (5.5) | 0.164 | 18 (19.6) | 27 (20.3) | 0.892 |
| CA19-9 post chemotherapy (kU/L), median (interquartile ranges) | 166 (56-541) | 141 (39-434) | 0.358 | 222 (63-641) | 222 (66-641) | 0.956 |
| Tumor size post chemotherapy (mm), mean (95 % CI) | 29 (26.5-31.1) | 26 (23.4-28.4) | 0.105 | 44 (39.9-47.3) | 40 (37.2-43.1) | 0.149 |
| Response Evaluation Criteria In Solid Tumours at restaging, n (%) |  |  | 0.199 |  |  | 0.831 |
| Complete/Partial response | 10 (10.9) | 7 (12.1) |  | 10 (10.8) | 13 (10) |  |
| Stable disease | 62 (67.4) | 45 (77.6) |  | 59 (64.2) | 80 (61.5) |  |
| Progressive disease | 20 (21.7) | 6 (10.3) |  | 23 (25) | 37 (28.5) |  |
| Surgical resection, n (%) | 45 (46.9) | 64 (67.4) | **0.004** | 12 (13) | 24 (18.1) | 0.314 |
| Types of procedures, n (%) |  |  | 0.620 |  |  | 0.517 |
| Pancreatoduodenectomy | 42 (93.3) | 58 (89.2) |  | 7 (58.3) | 18 (75) |  |
| Distal pancreatectomy | 2 (4.4) | 6 (9.2) |  | 1 (8.3) | 2 (8.3) |  |
| Total pancreatectomy | 1 (2.2) | 1 (1.5) |  | 4 (33.3) | 4 (16.7) |  |
| Concomitant vascular resection, n (%) | 26 (57.8) | 24 (36.9) | **0.031** | 8 (61.5) | 14 (56) | 0.743 |
| Postoperative complications ≥ Clavien grade 3, n (%) | 9 (9.4) | 11 (11.6) | 0.488 | 6 (50) | 8 (33.3) | 0.334 |
| Adjuvant chemotherapy, n (%) | 34 (75.6) | 50 (78.1) | 0.984 | 3 (25) | 12 (50) | 0.151 |
